# Trophic position and dietary breadth of bats revealed by nitrogen isotopic composition of amino acids

**DOI:** 10.1038/s41598-017-15440-3

**Published:** 2017-11-21

**Authors:** Caitlin J. Campbell, David M. Nelson, Nanako O. Ogawa, Yoshito Chikaraishi, Naohiko Ohkouchi

**Affiliations:** 1 University of Maryland Center for Environmental Science, Appalachian Laboratory, Frostburg, USA; 2 Department of Biology, Frostburg State University, Frostburg, USA; 3 Department of Biogeochemistry, Japan Agency for Marine-Earth Science and Technology, Yokosuka, Japan; 4 Institute of Low Temperature Science, Hokkaido University, Hokkaido, Japan

## Abstract

Bats perform important ecosystem services, but it remains difficult to quantify their dietary strategies and trophic position (TP) *in situ*. We conducted measurements of nitrogen isotopes of individual amino acids (*δ* ^15^N_AA_) and bulk-tissue carbon (*δ* ^13^C_bulk_) and nitrogen (*δ* ^15^N_bulk_) isotopes for nine bat species from different feeding guilds (nectarivory, frugivory, sanguivory, piscivory, carnivory, and insectivory). Our objective was to assess the precision of *δ* ^15^N_AA_-based estimates of TP relative to other approaches. TPs calculated from *δ* ^15^N values of glutamic acid and phenylalanine, which range from 8.3–33.1‰ and 0.7–15.4‰ respectively, varied between 1.8 and 3.8 for individuals of each species and were generally within the ranges of those anticipated based on qualitative dietary information. The *δ* ^15^N_AA_ approach reveals variation in TP within and among species that is not apparent from *δ* ^15^N_bulk_ data, and *δ* ^15^N_AA_ data suggest that two insectivorous species (*Lasiurus noctivagans* and *Lasiurus cinereus*) are more omnivorous than previously thought. These results indicate that bats exhibit a trophic discrimination factor (TDF) similar to other terrestrial organisms and that *δ* ^15^N_AA_ provides a reliable approach for addressing questions about variation in the TP of bats that have heretofore proven elusive.

## Introduction

Bats exhibit a diversity of feeding strategies, including nectarivory, frugivory, sanguivory, piscivory, carnivory, and insectivory. In doing so they carry out ecosystem services of ecological and socioeconomic importance, such as pollination and insect predation (e.g.^1,2^). Within and among these broad feeding guilds there exists variation in the extent to which different species are dietary specialists versus generalists (e.g.^3–6^). Knowledge of the dietary complexity and requirements of bats is important to assess their behavior, ecological and evolutionary processes, and susceptibility to extirpation or extinction (e.g.^7–11^). However, there remains limited understanding of how the dietary strategies of most organisms, including bats, vary across space and time in nature.

A primary reason for the lack of understanding of the dietary strategies of many species is the limitation of existing approaches for inferring dietary strategies. Direct observation and characterization of feeding behavior *in situ* is typically uncommon outside of experimental settings. Indirect assessments of animal diets are more common, but suffer from limitations. For example, morphological analysis of stomach contents or fecal material can provide precise dietary information. However, such approaches are labor-intensive, skewed toward detecting identifiable prey parts, and provide only a snapshot of a recent meal. DNA-based analyses of gut and/or fecal material can provide detailed dietary information (e.g.^12^), but also indicate only recently consumed resources and are typically non-quantifiable. Stable isotope ratios of carbon and nitrogen (*δ* ^13^C_bulk_ and *δ* ^15^N_bulk_, respectively) in animal tissues provide a more spatiotemporally integrated and inexpensive assessment of diet, and have become an important tool to enhance understanding of the prey items and trophic positions (TP) of wildlife, including bats (e.g.^4,13–16^). However, a challenge to interpreting such data in the context of TP is that the isotope values of a consumer’s tissues inherently reflect changes related to both the consumer’s TP and to the isotope values of the primary producers (autotrophs) at the base of the consumer’s food web, the latter of which can vary spatially and/or temporally and are often unknown^17^. This issue concerning interpretation of TP from *δ* ^15^N_bulk_ may be particularly important for mobile organisms, such as bats, that feed across broad spatial scales on potentially isotopically distinct food webs (e.g.^8,17,18^).

Analysis of *δ* ^15^N values of individual amino acids (*δ* ^15^N_AA_) emerged within the last ~15 years as a valuable tool for improving assessment of the trophic status of organisms in marine, freshwater, and terrestrial ecosystems (e.g.^18–22^). The basis of this method is that in certain amino acids (called “trophic” amino acids), the transamination and deamination reactions that form and cleave C-N bonds lead to isotopic fractionations and more positive *δ* ^15^N values at higher trophic levels. In contrast, C-N bonds are not created or broken during the metabolic processing of a few amino acids that are only biosynthesized by autotrophs (called “source” amino acids), which means that they confer little shift in *δ* ^15^N values across trophic levels^20,23^ and thus integrate the *δ* ^15^N values of the autotrophs eaten by consumers in food webs. Two common amino acids that are representative of trophic and source amino acids are glutamic acid and phenylalanine, respectively. Assuming similar turnover times (or periods of integration) for trophic and source amino acids, the TP of an organism can be estimated from its *δ* ^15^N values of glutamic acid (*δ* ^15^N_Glu_) and phenylalanine (*δ* ^15^N_Phe_) as

1 $${\rm{T}}{\rm{P}}=({\delta }^{15}{{\rm{N}}}_{{\rm{G}}{\rm{l}}{\rm{u}}}-{\delta }^{15}{{\rm{N}}}_{{\rm{P}}{\rm{h}}{\rm{e}}}+\beta )/{\rm{T}}{\rm{D}}{\rm{F}}+1$$

where β represents the difference between *δ* ^15^N_Glu_ and *δ* ^15^N_Phe_ in autotrophs, and TDF represents the trophic discrimination factor. β values depend on whether primary producers in food webs are aquatic or terrestrial (including C_3_ plants or agricultural C_4_ plants)^19^. Studies of terrestrial insects^19,24–26^; microorganisms^27^; and mammals, including modern^27,28^ and fossil^29,30^ herbivores, carnivores, and ancient humans^31,32^ suggest that a TDF of 7.6 ± 1.2‰ (1σ) is applicable for terrestrial organisms. However, relative to marine organisms, this tool has been applied to only a limited number of terrestrial taxa and its further use is likely to provide more quantitative and precise estimates of the TP of individuals of other species of ecologically important terrestrial organisms.

We measured *δ* ^13^C_bulk_ and *δ* ^15^N_bulk_, along with *δ* ^15^N_AA_, from nine bat species with relatively well-characterized and specialized diets and which represent a variety of feeding guilds. We use these data to assess the precision of *δ* ^15^N_AA_-based estimates of TP relative to *δ* ^15^N_bulk_ and estimates of TP inferred from known dietary information.

## Materials and Methods

### Species and samples

**Figure 1:**
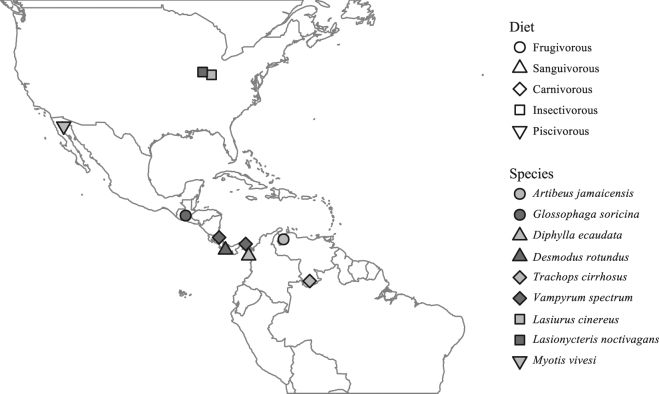
Locations where samples were obtained from 9 bat species. Map was generated in the R programming language v3.3.1 (R: A language and environment for statistical computing. v3.3.1, R Foundation for Statistical Computing, Vienna, Austria [2016] https://www.r-project.org/)^73^, using the “maps” package v3.2.0 (http://cran.r-project.org/package=maps)^85^ and public domain political boundary data published by Natural Earth (http://www.naturalearthdata.com/).

We obtained hair samples from two species of herbivorous bats, two species of sanguivorous bats, two species of insectivorous bats, one species of piscivorous bat, and two species of carnivorous bats in the Americas. All samples were obtained from dry skins of carcasses housed in the Smithsonian National Museum of Natural History’s Division of Mammals collection, with the exception of samples from carcasses of the insectivorous species, which were obtained from a wind-energy facility. For each species, all individuals were collected from the same location within North, Central, or South America, with the exception of individuals of *Vampyrum spectrum* that were obtained from two locations (Fig. 1, Table S1). We collected hair because, unlike other tissues that turnover continuously (e.g. blood), hair is metabolically inert following its growth. Therefore, hair isotopic values should reflect an integrated measure of diet during the period of hair growth^33^. Bats living in temperate regions are thought to molt during the summer months^34–38^, although there may be variation in the timing of molt between sexes and among age groups. Furthermore, the timing of annual molt in neotropical bats is poorly understood^34^.

Basic dietary information is known for each species from which we obtained hair. Although the diets of these species are better understood than those of most bat species, such information is not quantitative. Furthermore, understanding of the diets and TPs of many of the organisms that these species of bats prey upon is lacking. Such uncertainties make it challenging to use existing dietary information from the literature to precisely estimate the expected TP of each species. Nevertheless, broad differences in TP are expected, such as that herbivorous bats eat at lower TPs than carnivorous bats.

We obtained hair from the following species:

- Jamaican fruit bat (*Artibeus jamaicensis*), a frugivore that lives in Mexico, Central America, and far northwestern South America that is known to eat fruit and occasionally leaves and flowers^14,39,40^.
- Pallas’s long-tongued bat (*Glossophaga soricina*), a nectarivore and frugivore found in Central and South America^41^ that is also known to prey upon insects^16,42,43^.
- Hairy-legged vampire bat (*Diphylla ecaudata*), a sanguivore occurring in Mexico, Central America, and South America that consumes blood, mostly or entirely of small birds^44,45^. Blood contains a large proportion of non-metabolized amino acids derived from food amino acids and peptides^28,46^, and thus *D*. *ecaudata*’s TP should be similar to that of its prey rather than being higher than its prey.
- Common vampire bat (*Desmodus rotundus*), a sanguivore with a distribution that includes Mexico, Central America, and South America. Isotopic data suggests this species prefers to ingest blood from cattle^47^. Like *D*. *ecaudata*, we expect the TP of *D*. *rotundus* to be similar to that of its prey, but perhaps lower, because *D*. *rotundus* feeds exclusively on blood from herbivores whereas *D*. *ecaudata* may also feed on organisms at higher trophic levels.
- Fringe-lipped bat (*Trachops cirrhosus*), a carnivore that lives in southern Mexico, Central America, and South America that eats a diversity of prey, including insects, small birds, small mammals (including rodents and small bats), and lizards^48,49^.
- Spectral bat (*Vampyrum spectrum*), a carnivore from southern Mexico, Central America, and northern South America that eats small birds and mammals^48,50^.
- Hoary bat (*Lasiurus cinereus*), an insectivore found throughout North, Central, and South America that eats moths and other insects^51–55^. Prior studies indicate that plant material has occasionally been found in the stomach contents or fecal material of insectivorous bats^56^, including *L*. *cinereus* and *L*. *noctivagans* ^57–60^.
- Silver-haired bat (*Lasionycteris noctivagans*), an insectivore that occurs in North America that eats a variety of prey^51,52^.
- Fish-eating myotis (*Myotis vivesi*), a piscivore found around the Gulf of California that eats small marine fish (in the Engraulidae, Clupeidae, and Myctophidae families) and surface-swimming crustaceans. It is also thought to occasionally consume terrestrial insects^61,62^.

### Bulk isotope analysis

We obtained hair from 5–13 individuals per species. These samples were cleaned using 1:200 Triton X-100 detergent and 100% ethanol, rinsed 5 times with nano-pure water, and air dried to remove any potential oil or contaminants from the surface of the hair^63^. Approximately 1 mg of cleaned hair was analyzed for *δ* ^13^C and *δ* ^15^N using a Carlo Erba NC2500 elemental analyzer (CE Instruments, Milano, Italy) interfaced with a ThermoFinnigan Delta V+ isotope ratio mass spectrometer (IRMS; Bremen, Germany) at the Central Appalachians Stable Isotope Facility (CASIF) at the Appalachian Laboratory (Frostburg, Maryland, USA). The *δ* ^13^C and *δ* ^15^N data were normalized to the VPDB and AIR scales, respectively, using a two-point normalization curve with laboratory standards calibrated against USGS40 and USGS41. The among-run precision of a keratin standard analyzed multiple times alongside samples was 0.1‰ for *δ* ^13^C and *δ* ^15^N.

### Amino-acid δ^15^N analysis

The preparation of samples for *δ* ^15^N_AA_ analysis is time consuming and costly relative to *δ* ^13^C_bulk_ and *δ* ^15^N_bulk_ analysis. Thus, for *δ* ^15^N_AA_ we selected a subset of the individuals that were analyzed for *δ* ^13^C_bulk_ and *δ* ^15^N_bulk_. We performed *δ* ^15^N_AA_ analysis on cleaned hair from five individuals of *M*. *vivesi* and three individuals for all other species. We selected individuals spanning a range of *δ* ^15^N_bulk_ values to use for *δ* ^15^N_AA_.

Nitrogen isotope analysis of amino acids was conducted at the Department of Biogeochemistry at the Japan Agency for Marine-Earth Science and Technology (JAMSTEC; Yokosuka, Kanagawa Prefecture, Japan). Samples were prepared as in Chikaraishi *et al*.^18,23^. Briefly, ~1 mg of hair from each sample was hydrolyzed, delipified, and derivatized; the derivatives were then extracted. Compound-specific nitrogen isotope analysis was conducted with an Agilent 6890N gas chromatograph coupled to a ThermoFinnigan Delta Plus XP IRMS through GC combustion III interface (Bremen, Germany). Any potential chemicals that hair may have been exposed to (e.g. during preservation as museum specimens) are unlikely to cleave or form bonds associated with the amino-group nitrogen of amino acids and thus are unlikely to affect *δ* ^15^N_AA_ (e.g.^64^). We compared *δ* ^15^N_AA_ values from hair cleaned as above vs. rinsed only with water from a subset of individuals, and we observed no effect of the cleaning procedure on the relative abundance of amino acids or *δ* ^15^N_AA_ values (data not shown).

### Data analysis

The TP of each individual was calculated using equation 1 with the measured *δ* ^15^N_Glu_ and *δ* ^15^N_Phe_ values, the assigned β values, and the TDF value (7.6 ± 1.2‰, 1σ) recommended for terrestrial organisms^19,24–27^. Ideally, TDF values are assessed empirically for organisms of interest via controlled-diet studies (e.g.^18,65,66^). However, such studies are challenging to perform for taxa, such as bats, that are generally difficult to rear in captivity on diets limited to represent a specific TP. Therefore, we used a TDF value of 7.6 ± 1.2‰, which is thought to be applicable for terrestrial food webs (e.g.^19,24,26,27,29^), for bats with different feeding strategies that have relatively well-characterized and specialized diets.

**Figure 2:**
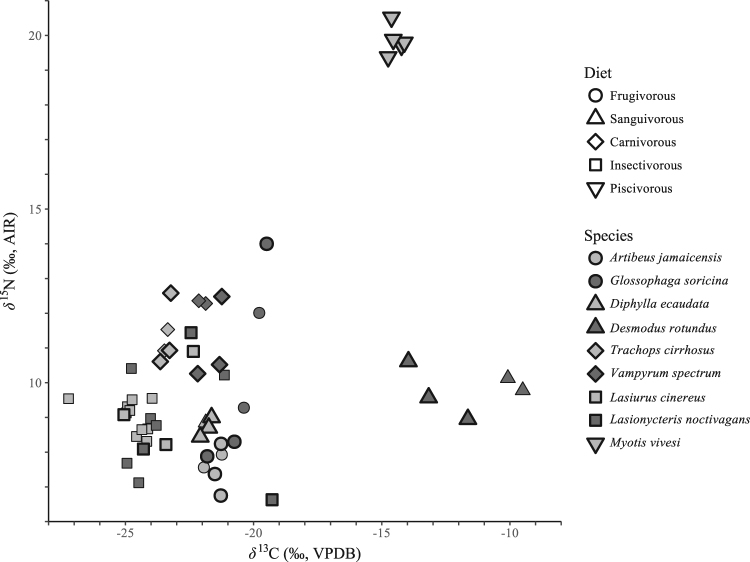
*δ* ^13^C and *δ* ^15^N values of bulk hair samples. Samples selected for amino-acid *δ* ^15^N analysis are outlined in bold.

Values of β differ among aquatic plants, C_3_ plants, and C_4_ plants^19^. Thus, we used *δ* ^13^C_bulk_ and *δ* ^15^N_bulk_ data to identify bats eating on food webs supported by these groups of primary producers (e.g.^67–69^) and then assign appropriate β values. Individuals with hair *δ* ^13^C values > −19‰ and *δ* ^15^N values > 12‰, the approximate thresholds for identifying individuals using marine-based food webs^67,68^, were considered to consume marine prey, and thus were assigned a β value for primary producers in aquatic systems of −3.4 ± 0.9‰ (1σ). Individuals with *δ* ^13^C values > −15‰ and *δ* ^15^N values < 12‰ were presumed to be eating on C_4_-plant based terrestrial food webs and thus were assigned a β value of −0.4 ± 1.7‰ (1σ). All other individuals were assigned a β value for terrestrial C_3_ plants of +8.4 ± 1.6‰ (1σ)^70^. For species in food webs that include aquatic and terrestrial, or C_3_ and C_4_, plants it is possible to use *δ* ^13^C_bulk_ data to calculate a “mixed” β value^71^, but given the relatively specialized diets of the species we analyzed and the strong observed separation of *δ* ^13^C_bulk_ and *δ* ^15^N_bulk_ values (Fig. 2) we did not use such an approach in this study.

The uncertainties in *δ* ^15^N_Glu_ and *δ* ^15^N_Phe_ values (±0.5‰, 1σ), β values (as above), and TDF (as above), were propagated in equation 1 using the “propagate” package (version 1.0–4) in R (version 3.3.1) to assess uncertainty in calculated TP values^72^. Analysis of variance (ANOVA) of the calculated TP of each species, as well as *δ* ^15^N_bulk_ for the samples from which *δ* ^15^N_AA_ measurements were made, was performed in R^73^.

## Results

Amongst all species, *δ* ^13^C_bulk_ ranges between −27.2 and −9.5‰, and *δ* ^15^N_bulk_ between 6.6 and 20.5‰. Samples from *M*. *vivesi* have *δ* ^13^C_bulk_ > −15‰ and *δ* ^15^N_bulk_ > 19‰, and samples from *D*. *rotundus* have *δ* ^13^C_bulk_ > −15‰ and *δ* ^15^N_bulk_ < 11‰. Thus, β values of −3.4 ± 0.9‰ and −0.4 ± 1.7‰ are used in calculations of the TP for individuals of these species, respectively. All other individuals have *δ* ^13^C_bulk_ < −19‰ and *δ* ^15^N_bulk_ < 12‰ and thus a β value for terrestrial food webs (+8.4 ± 1.6‰) is used in calculating the TP of the remaining seven species (Fig. 2).

Mean *δ* ^15^N_bulk_ is indistinguishable between *A*. *jamaicensis* and *D*. *rotundus*, *D*. *ecaudata*, *L*. *cinereus* and *L*. *noctivagans* (Fig. S1). Mean *δ* ^15^N_bulk_ is highest for *M*. *vivesi*, followed by *T*. *cirrhosus* and *V*. *spectrum*. Mean *δ* ^15^N_bulk_ of *T*. *cirrhosus* and *V*. *spectrum* overlaps with those of *D*. *rotundus* and *G*. *soricina*.

**Figure 3:**
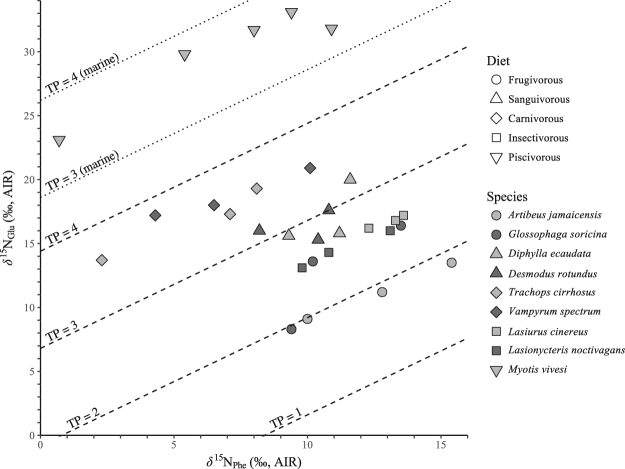
*δ* ^15^N values of glutaminic acid and phenylalanine. Dashed and dotted lines denote trophic position (TP) for terrestrial and marine systems, respectively.

**Figure 4:**
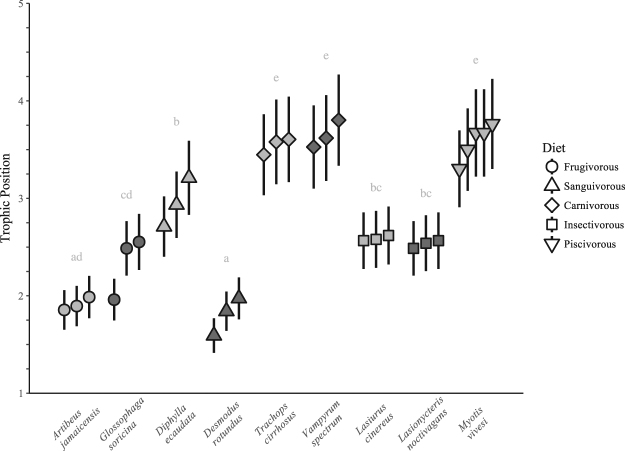
Trophic position of each individual calculated from amino-acid *δ* ^15^N values. Points and error bars denote first-order Taylor expansion mean and one standard deviation, respectively. Letters indicate species-level differences in trophic position determined by Tukey’s test of mean comparison.

**Figure 5:**
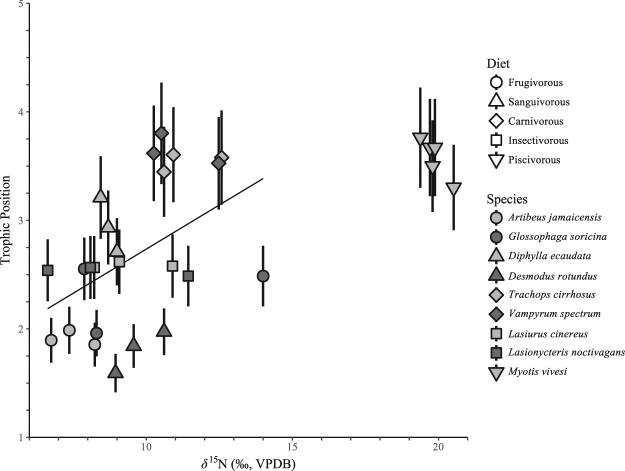
Relationship between bulk δ^15^N values of hair and trophic position of each individual calculated from amino-acid δ^15^N values. The regression line (r^2^ = 0.18, p = 0.023, n = 24) is fit through all of the data, excluding *M. vivesi*.

Across all species, *δ* ^15^N_Glu_ values range between 33.1 and 8.3‰, and δ^15^N_Phe_ values range between 15.4 and 0.7‰ (Fig. 3). *M*. *vivesi* has the largest range of variation of *δ* ^15^N_Phe_ values (0.7–10.9‰). The calculated TP values are highest for individuals of the piscivorous species (*M*. *vivesi*, 3.3–3.8) and the carnivorous species (*V*. *spectrum*, 3.5–3.8; *T*. *cirrhosus*, 3.4–3.8). The calculated TP values are lowest for *A*. *jamaicensis* (1.9–2.0) and *D*. *rotundus* (1.6–2.0). The calculated TP values of *G*. *soricina* (2.0–2.6) are not distinct from *A*. *jamaicensis*. The calculated TP values for *D*. *ecaudata* (2.7–3.2) are higher than those of the *A*. *jamaicensis*, *G*. *soricina*, and *D*. *rotundus*, but indistinct from those of the insectivorous species, *L*. *cinereus* (2.6) and *L*. *noctivagans* (2.5–2.6). The calculated TP values for the insectivorous species are higher than those of *A*. *jamaicensis* and *D*. *rotundus*, but indistinct from *G*. *soricina* and *D*. *ecaudata* (Fig. 4). There is a positive relationship (r^2^ = 0.18, p = 0.023, n = 24) between *δ* ^15^N_bulk_ and TP of each individual calculated from *δ* ^15^N_AA_ (Fig. 5).

## Discussion

The broad patterns of variation in bulk-tissue isotopic values reflect the variation expected from known dietary information. For example, *M*. *vivesi* had high *δ* ^13^C_bulk_ and *δ* ^15^N_bulk_ values, which is consistent with the facts that *M*. *vivesi* is a piscivore and *δ* ^13^C and *δ* ^15^N values are typically high in aquatic-based food chains^67,68^. The less negative *δ* ^13^C values of *D*. *rotundus* supports a prior study suggesting that this species prefers to ingest blood from cattle that, in contrast to native mammals that are part of C_3_-plant based foodwebs, are typically fed an agricultural C_4_-plant (i.e. corn) based diet in Central America where our *D*. *rotundus* samples originate^47^. However, beyond assisting with coarse dietary characterization (e.g. marine vs. terrestrial or C_3_ vs. C_4_), we are hesitant to use our *δ* ^15^N_bulk_ data to assess the TP of the species we analyzed because the baseline *δ* ^15^N values of their foodwebs are unknown. The species with the highest expected and calculated TPs in our dataset (*M*. *vivesi*, *V*. *spectrum* and *T*. *cirrhosus*) had some of the lowest *δ* ^15^N_Phe_ values (Figs 3 and 4). Low basal *δ* ^15^N values of the foodwebs on which these species ate likely depress their *δ* ^15^N_bulk_ values relative to those otherwise expected for organisms eating at relatively high TPs. Indeed, the *δ* ^15^N_bulk_ values of the carnivorous species were indistinct from those of a frugivore, *G*. *soricina*, and a sanguivore, *D*. *rotundus* (Figs 2, 5 and S1).

TP values calculated using the *δ* ^15^N_AA_ approach are broadly similar to differences in TP expected based on qualitative information about the diets of each species, which helps to validate the *δ* ^15^N_AA_ approach for identifying the TP of bats. Strictly herbivorous animals provide perhaps the best opportunity to evaluate the effectiveness of *δ* ^15^N_AA_ for identifying TP. In this regard, the calculated TP values for *A*. *jamaicensis* (1.9–2.0) are what would be anticipated for a strict frugivore. One individual of *G*. *soricina* had a TP of 2.0, but two other individuals of *G*. *soricina* had a TP of 2.5–2.6. Such inter-species differences may reflect that *A*. *jamaicensis* is thought to be a strict frugivore, whereas *G*. *soricina* is thought to also prey upon insects and thus be relatively more omnivorous^16,42,43^. However, because the timing of molt of these species is not well defined, we cannot exclude the potential that such variation could also indicate differences in TP through time related to hair growth potentially occurring during different time periods among individuals. Furthermore, as expected, TP values were highest for the piscivorous and carnivorous species that are not thought to directly consume primary producers. Calculated TP values were overall lowest for the frugivorous species, as well as one sanguivore, *D*. *rotundus*. The mean TP calculated from the *δ* ^15^N_AA_ approach for the other sanguivore, *D*. *ecaudata* was roughly one TP higher than *D*. *rotundus*, likely because *D*. *rotundus* feeds exclusively on blood from herbivores, whereas *D*. *ecaudata* also feeds on blood of animals at higher trophic levels^44,45,47^. Such differences in TP between *D*. *rotundus* and *D*. *ecaudata* were not apparent in *δ* ^15^N_bulk_ (Figs 5 and S1). Together, such results illustrate the effectiveness of *δ* ^15^N_AA_ data for determining the TP of bats across diverse dietary groups.

Insectivores should have a TP of ≥3.0. Thus, our finding of a relatively low TP (2.5–2.6) for the analyzed samples of *L*. *noctivagans* and *L*. *cinereus* was unexpected. One factor by which the TP of these insectivorous bats may have been underestimated is if the TDF value of 7.6 ± 1.2‰ was too large. Although TDF displays minor variation in terrestrial organisms, greater variation in TDF exists in aquatic organisms likely in response to differences in mode of nitrogen excretion and diet quality (the amino acid composition of diet relative to the needs of a consumer)^74,75^. Mammals excrete urea and thus any influence of mode of nitrogen excretion on the TDF value is likely to affect all bats similarly and is unlikely to explain these results. In aquatic settings, when dietary protein content is low (e.g. for herbivores) TDF is generally high and when protein content is high (e.g. carnivores) TDF values are generally lower^74,75^. If the TDF value used for *L*. *noctivagans* and *L*. *cinereus* was too large, perhaps because of a diet potentially high in protein, then TP could be underestimated. However, the TPs calculated from *δ* ^15^N_AA_ values for other bat species of higher (and lower) TP appear reasonable, which suggests that it is unlikely that TPs derived from *δ* ^15^N_AA_ values would be consistently less than 3.0 for bats that eat insects exclusively. Furthermore, it is unlikely that the calculated TPs of *L*. *noctivagans* and *L*. *cinereus* were driven lower by undigested plant material that may have been in the alimentary canals of their insect prey, as prior studies based on *δ* ^15^N_AA_ data indicate that herbivorous insects consistently have a calculated TP of 2.0^18,20,24^.

Prior studies indicate that small quantities of plant material are occasionally found in the stomach contents or fecal material of insectivorous bats^56^, including *L*. *noctivagans* and *L*. *cinereus* ^57–60^. The exoskeletons of insects are highly resistant and thus more easily digested plant material may be less likely encountered during morphological analysis of gut or fecal material than insect remains (e.g.^76^). If so, the small quantities of identifiable plant material found in prior studies could suggest that these species are more omnivorous, with greater dietary flexibility to consume plant-based material, than morphological assessments of stomach contents or fecal material suggest.

Although *L*. *noctivagans* and *L*. *cinereus* had nearly identical TP based on *δ* ^15^N_AA_, *δ* ^15^N_bulk_ of *L*. *noctivagans* varied about twice as much as *L*. *cinereus* (Figs 2 and 5). The samples from these species were obtained from individuals at the same location and their hair is thought to molt during the summer months^33^. Therefore, these results may suggest that *L*. *noctivagans* has a more general diet and consumes a greater variety of prey (that differ in *δ* ^15^N at the base of their food webs) than does *L*. *cinereus*, although both species eat at similar TPs. Prior studies suggest that *L*. *noctivagans* and *L*. *cinereus* hunt a diversity of prey, although other studies consider them to specialize on moths^51,52^. Although our sample size is small, our results suggest that *L*. *noctivagans* may be more of a generalist than *L*. *cinereus*. Consistent with this idea, *L*. *cinereus* uses narrow-band (long-range) echolocation calls, which might indicate that it is able to be relatively selective about the prey it detects and captures since it detects them from far away. In contrast, *L*. *noctivagans* uses broad-band (short-range) echolocation calls that perhaps provide less time for it to decide which prey to pursue and therefore less dietary specialization^51^. Future studies should further investigate the degree of omnivory and dietary specialization exhibited by these and other species of insectivorous bats.

We observed a particularly wide range of variation (i.e. 0.7–10.9‰) in *δ* ^15^N_Phe_ values of *M*. *vivesi*. This range suggests differences in the *δ* ^15^N values of primary producers at the base of the food webs upon which *M*. *vivesi* feeds. Since all samples of this species were obtained on the same day and year it is unlikely that such variation is related to seasonal and/or inter-annual variation in an environmental factor such as climate. Rather, we speculate that such variation may be related to spatial gradients in the extent of denitrification and nitrogen fixation in marine waters of the Gulf of California region (e.g.^77,78^). Such gradients might allow some of the prey that *M*. *vivesi* eats to originate from waters with primary producers with relatively low *δ* ^15^N values (e.g. where nitrogen fixation is extensive) and high *δ* ^15^N values (e.g. where denitrification is extensive). Alternatively, some of the prey that *M*. *vivesi* eats (e.g. Clupeidae) may originate from areas further north in the Pacific Ocean, where *δ* ^15^N values are lower, and then transport such low *δ* ^15^N values in their biomass to the Gulf of California region.

The error bars in the TP calculated for each individual using the *δ* ^15^N_AA_ approach are small, which illustrates the relatively precise estimates of TP for individual organisms that are possible to obtain from *δ* ^15^N_AA_. The positive relationship between *δ* ^15^N_bulk_ and TP calculated from *δ* ^15^N_AA_ for the species eating on terrestrial food webs is not unexpected because *δ* ^15^N_bulk_ is partly influenced by TP. However, the predictive capacity of *δ* ^15^N_bulk_ for estimating TP calculated from *δ* ^15^N_AA_ is weak. For example, *δ* ^15^N_bulk_ of individuals of *L*. *noctivagans* span a range of ~4.8‰, but exhibit variation of only 0.1 in their TP as calculated from *δ* ^15^N_AA_ (Fig. 5). Furthermore, *δ* ^15^N_bulk_ values are indistinct among some species with known dietary differences and TPs as inferred from *δ* ^15^N_AA_, such as *G*. *soricina* and the carnivorous species (Fig. S1).

Overall, our results indicate that *δ* ^15^N_AA_ values are useful for assessing variation in the TP of bats. Therefore, *δ* ^15^N_AA_ data will be helpful in addressing ecological and evolutionary questions that have previously been difficult to answer. For example, omnivory is thought to be less common in insectivores than frugivores^79,80^, and *δ* ^15^N_AA_ data may be used to characterize the degree of omnivory in such bat species more precisely than is possible using other approaches. Furthermore, nearly half of all bat species are endangered, threatened, or of conservation concern^81^. Bats are particularly susceptible to extirpation and extinction because of their low annual reproductive rates^82^, and bat species of conservation concern are thought to have relatively specialized diets^7^. Thus, *δ* ^15^N_AA_ data could also be used to assess variation in the degree of dietary specialization (measured as shifts in TP) of populations, species, and/or communities and thus identify those at relatively greater risk. Finally, such data could be used to test ecological theory, which predicts dietary specialists to be most prevalent where food availability is stable, whereas generalists are predicted to thrive in environments where resource availability varies (e.g.^10,83,84^).

## Electronic supplementary material

Supplementary Figure S1

Supplementary Table S1
