# Supplementary material for: Trophic position and dietary breadth of bats revealed by nitrogen isotopic composition of amino acids
Source: Sci Rep. 2017 Nov 21;7:15932. doi: 10.1038/s41598-017-15440-3 (PMC5698291; doi:10.1038/s41598-017-15440-3)
Supplement: Supplementary file 1 — Supplementary Figure S1 [file 41598_2017_15440_MOESM1_ESM.pdf]

Trophic position and dietary breadth of bats revealed by nitrogen isotopic composition of amino acids

Caitlin J. Campbell *, David M. Nelson, Nanako O. Ogawa, Yoshito Chikaraishi, and Naohiko Ohkouchi

Supplemental Information 1

Supplementary Figure 1

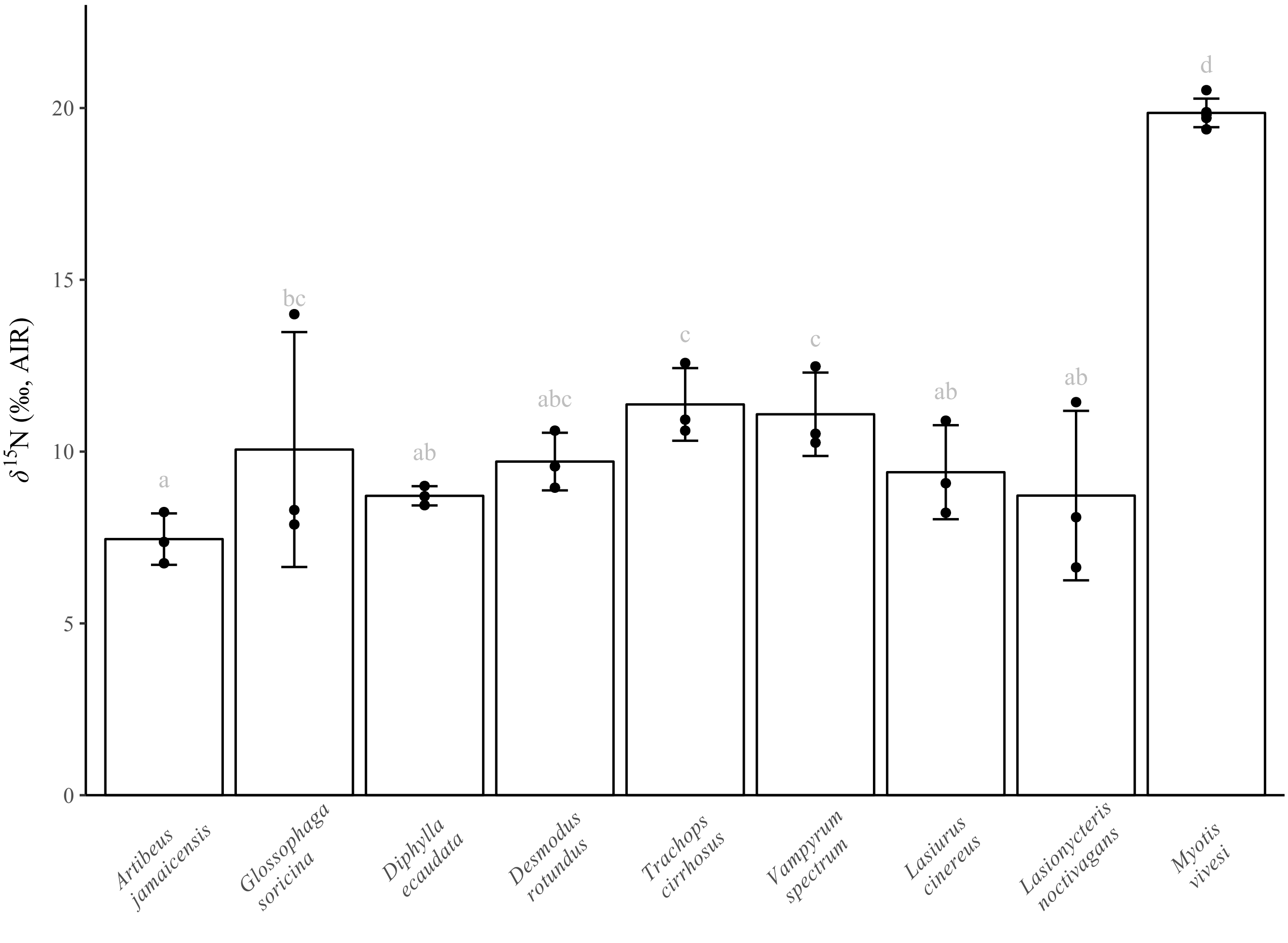

Sup. Fig. 1 δ^{15}N values of bulk hair samples. Letters indicate species-level differences in δ^{15}N values determined by Tukey's test of mean comparison.
